# Synthesis and application of trifluoroethoxy-substituted phthalocyanines and subphthalocyanines

**DOI:** 10.3762/bjoc.13.224

**Published:** 2017-10-27

**Authors:** Satoru Mori, Norio Shibata

**Affiliations:** 1 Department of Nanopharmaceutical Sciences, Nagoya Institute of Technology, Gokiso, Showa-ku, Nagoya 466-8555, Japan; 2 Department of Life Science and Applied Chemistry, Nagoya Institute of Technology, Gokiso, Showa-ku, Nagoya 466-8555, Japan

**Keywords:** aggregation, fluorine, phthalocyanine, subphthalocyanine, trifluoroethoxy

## Abstract

Phthalocyanines and subphthalocyanines are attracting attention as functional dyes that are applicable to organic solar cells, photodynamic therapy, organic electronic devices, and other applications. However, phthalocyanines are generally difficult to handle due to their strong ability to aggregate, so this property must be controlled for further applications of phthalocyanines. On the other hand, trifluoroethoxy-substituted phthalocyanines are known to suppress aggregation due to repulsion of the trifluoroethoxy group. Furthermore, the electronic characteristics of phthalocyanines are significantly changed by the strong electronegativity of fluorine. Therefore, it is expected that trifluoroethoxy-substituted phthalocyanines can be applied to new industrial fields. This review summarizes the synthesis and application of trifluoroethoxy-substituted phthalocyanine and subphthalocyanine derivatives.

## Introduction

Phthalocyanines [1–3] are analogues of porphyrin condensed with four isoindoline units via a nitrogen atom and exhibit a deep blue color due to their wide 18π electron conjugation. Among them, the most fundamental phthalocyanine copper complex, which does not have a substituent on its periphery, is known as a blue organic pigment called phthalocyanine blue [4–7]. Phthalocyanines have long been used as a blue pigment for, e.g., road signs and bullet trains because they can be manufactured cheaply, are very robust and are difficult to discolor [8]. Conventional pigments are intended for the use as colorants, so only coloring characteristics such as color tone, stability, solubility and others, have been regarded as being important. However, as the development of optoelectronics advanced in recent years, the term “functional dyes” was proposed [9–11]. Functional dye is a generic name for dyes that exceed the framework of coloration and show various chemical and physical responses. In modern times, unlike the concept of colorants in the past, dyes have been developed for various applications based on their unique functions. Phthalocyanines are also expected to be applied as functional dyes. A series of compounds having a macrocyclic π-conjugated system including porphyrin are known as functional dyes. In particular, phthalocyanines have a wide range of functions and are expected to serve as novel material in the future. Besides blue organic pigments such as ink and colorants, it is expected to be widely applied to electronic devices in the medical field such as color filters for liquid crystal screens [12–13], storage media [14], dye-sensitized solar cells [15–16], photodynamic cancer treatment method [17] and other applications.

Incidentally, fluorine is one of the elements that is expected to improve the state-of-the-art science technology [18]. Fluorine is located at the top right of the periodic table of elements excluding the noble gas elements of group 18. The size of fluorine is approximately 23% larger than hydrogen and has the highest electronegativity (4.0) among all the elements [19–20]. Therefore, substituting fluorine for a single hydrogen atom in the structure of an organic compound may cause noticeable changes in the dipole moment although the change in the chemical structure is small [21–22]. As a result, compounds into which fluorine has been introduced may show beneficial changes in their properties. In addition, among the chemical bonds formed by carbon, fluorine bonds have the highest binding energy [23], such that fluorine-containing compounds often show resistance to metabolism and oxidation compared to non-fluorinated compounds. In addition to their stability, perfluorinated compounds exhibit non-tackiness, low friction, and the ability to repel water and oil [24]. Therefore, they are used as functional materials such as fluorocarbon polymer. Fluorine is also an important key element in the fields of medical and agricultural chemistry [25–31]. As described above, fluorine is considered an essential element for next-generation science technology, and many researchers have studied methods for the synthesis of fluorine-containing compounds.

**Scheme 1:**
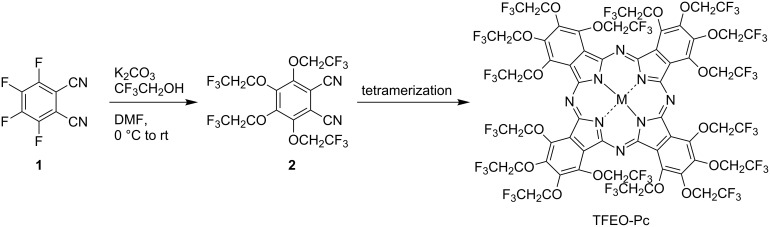
Synthesis of trifluoroethoxy-substituted phthalocyanine.

By introducing a fluorine atom, phthalocyanines are also expected to become novel functional materials that reflect the specific properties of the fluorine atom. Fluorine-containing phthalocyanines have been found to behave differently from non-fluorine phthalocyanines due to the specific properties of fluorine [32]. For example, since phthalocyanines have a conjugate planar structure with high symmetry, it is very difficult to dissolve them in ordinary organic solvents. For this reason, fluorine is often introduced to improve the solubility of phthalocyanine in organic solvents [33–35]. Although ordinary phthalocyanines are known to be slightly soluble compounds, fluorine-containing phthalocyanines show high solubility in various organic solvents. Furthermore, they are thermally and chemically stable due to a strong carbon–fluorine bond [36–38]. Moreover, due to the strong electronegativity of fluorine, fluorinated phthalocyanines show interesting electrochemical behavior [39–40]. As described above, since the properties of fluorine-containing phthalocyanines change considerably due to the strong electronegativity of fluorine, their development as unprecedented new functional materials is expected. Among other fluorine-containing phthalocyanines, trifluoroethoxy-substituted phthalocyanines (TFEO-Pcs) having a trifluoroethoxy group (-OCH_2_CF_3_) at the periphery of the phthalocyanine can be conveniently synthesized from trifluoroethanol (CF_3_CH_2_OH). First, tetrakis(trifluoroethoxy)phthalonitrile (**2**) is synthesized by reacting commercially available tetrafluorophthalonitrile (**1**) with trifluoroethanol. Thereafter, tetramerization is carried out as a general synthesis of phthalocyanine to induce TFEO-Pc (Scheme 1). Since TFEO-Pcs have a large number of fluorine atoms, their solubility in organic solvents is extremely high. Therefore, TFEO-Pcs can be easily purified by silica gel column chromatography. In addition, when a phthalocyanine is substituted with an electron-donating group, such as an alkoxy group, the electron density in the phthalocyanine macrocycle increases, making it easily oxidized and becoming unstable. On the other hand, TFEO-Pcs are compounds that are stable and easy to handle because the electron density in the macrocycle does not increase due to the strong electron-withdrawing action of the fluorine atoms. By utilizing the robustness and high solubility of TFEO-Pcs, it is possible to synthesize various phthalocyanine derivatives that are difficult to synthesize conventionally due to the weak solubility of phthalocyanine. Due to the strong electronegativity of the fluorine atom, an electron-deficient π-conjugated system is formed, so it is expected that phthalocyanine, which originally acts as an electron donor, will exhibit an electron acceptor property. Thus, it is expected that TFEO-Pcs can be developed as unique functional molecules [41]. This review summarizes the synthesis of various TFEO-Pc derivatives and the effect of fluorine on their properties.

## Review

### Properties of trifluoroethoxy-substituted phthalocyanines

Research on TFEO-Pc has taken place since the 1990s [42]. The early stages of this research focused on spectroscopic investigations of metal-free phthalocyanine and its zinc complex which showed the most fundamental spectroscopic properties among the phthalocyanines [43–44]. Trifluoroethoxy-substituted zinc phthalocyanine (TFEO-ZnPc) shows a very strong absorption peak called the Q band in the longer wavelength region greater than 700 nm. It is red-shifted compared with the unsubstituted phthalocyanine zinc complex due to the influence of the oxygen atom directly connected to the phthalocyanine macrocycle. The phenomenon of solvatochromism [45–46] is defined by the polarity of the solvent, and the Q band is shifted to a shorter wavelength region in a highly polar solvent. In addition to the Q band, in solvatochromism, a small absorption peak forms on the slightly shorter wavelength side than the Q band which is caused by vibrational progression of the Q band and a broad peak referred to as the Soret band in the 300–400 nm region. Unlike the strong absorption of the Q band, the emission of fluorescence by TFEO-ZnPc is not very strong, and the Stokes shift is small, about 10 nm. On the other hand, metal-free trifluoroethoxy-substituted phthalocyanine (TFEO-H_2_Pc) shows a split in the Q band due to its low symmetry [47]. In metal-free phthalocyanine, the four pyrrole units in the center of the macrocycle have two protons. Therefore, metal-free phthalocyanine has the ability to donate and accept protons [48–50]. The protonation/deprotonation of the nitrogen atom in phthalocyanines easily modifies its chemical and electronic properties. And this protonation/deprotonation phenomenon is expected to be applied to chemical sensors [51]. Due to the strong electronegativity of fluorine, the acidity of these pyrrole rings is increased in TFEO-H_2_Pc. For this reason, it is susceptible to deprotonation, whereas protonation hardly occurs.

Trifluoroethoxy-substituted nickel phthalocyanine (TFEO-NiPc) and trifluoroethoxy-substituted iron phthalocyanine (TFEO-FePc) were compared with non-fluorinated alkoxy-substituted phthalocyanines [52]. A slightly bathochromic shift of the Q band was observed for TFEO-Pcs relative to non-fluorinated alkoxylated phthalocyanines. Although the melting points of non-fluorinated alkoxylated phthalocyanines are lower than 200 °C, TFEO-NiPc does not melt below 300 °C. Phthalocyanine iron complexes, which are substituted by non-fluorinated alkoxy groups at all peripheries, are easily oxidized and unstable, so these cannot even be synthesized. On the other hand, TFEO-FePc is stabilized by the strong electronegativity of fluorine and has been successfully isolated. In measurements of cyclic voltammetry, iron phthalocyanines with an alkoxy group show a lower first oxidation potential than unsubstituted iron phthalocyanine, but TFEO-FePc shows a more difficult oxidation process than unsubstituted iron phthalocyanine. Furthermore, it is known that iron phthalocyanines easily form a μ-oxo dimer [53] that is connected face-to-face via oxygen, but a spectroscopic investigation revealed that it is difficult to form when using TFEO-FePc. This remarkable stability of TFEO-FePc is due to the low energy of the highest occupied molecular orbital (HOMO) [54] caused by the strong electronegativity of fluorine.

A noteworthy feature of the compounds od the TFEO-Pc series is their high solubility in various organic solvents. This high solubility is suitable for the investigation of the spectroscopic properties of phthalocyanines. TFEO-Pc is soluble not only in general organic solvents but also in liquid carbon dioxide (CO_2_) and supercritical CO_2_ [55]. These forms of CO_2_ have attracted attention as solvents which do not discharge volatile organic compounds (VOCs), and are expected to replace organic solvents as a countermeasure to VOC emissions [56–58]. Phthalocyanines that show solubility in CO_2_ are useful for industrial applications because they can be applied without using an organic solvent [59–60]. The plus and minus signs shown in Table 1 express solubility, where (+) means readily soluble, (−) means sparingly soluble, (++) means very high solubility, and (±) means moderate solubility. After conducting solubility studies on various phthalocyanines, only phthalocyanine in which all positions were substituted with trifluoroethoxy groups showed high solubility in both liquid CO_2_ and supercritical CO_2_. On the other hand, phthalocyanine without any trifluoroethoxy groups such as *tert*-butyl-substituted zinc phthalocyanine (*t*-Bu-ZnPc) or perfluorinated zinc phthalocyanine (F-ZnPc), showed low solubility in both forms of CO_2_ while phthalocyanine with zinc as the central metal tended to have higher solubility than metal-free phthalocyanine.

**Table 1:** Solubility of phthalocyanines in liquid CO_2_ and supercritical CO_2_.

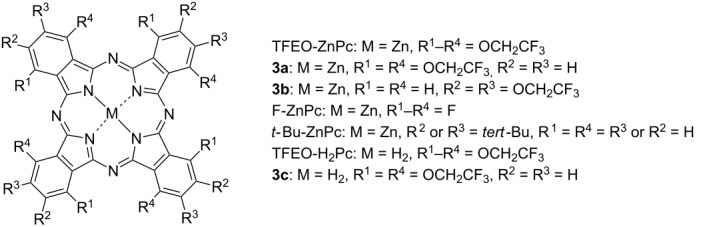		

compound	in liquid CO_2_	in supercritical CO_2_

TFEO-ZnPc	++	+
**3a**	+	–
**3b**	±	–
F-ZnPc	–	–
*t*-Bu-ZnPc	–	–
TFEO-H_2_Pc	+	–
**3c**	±	–

**Scheme 2:**
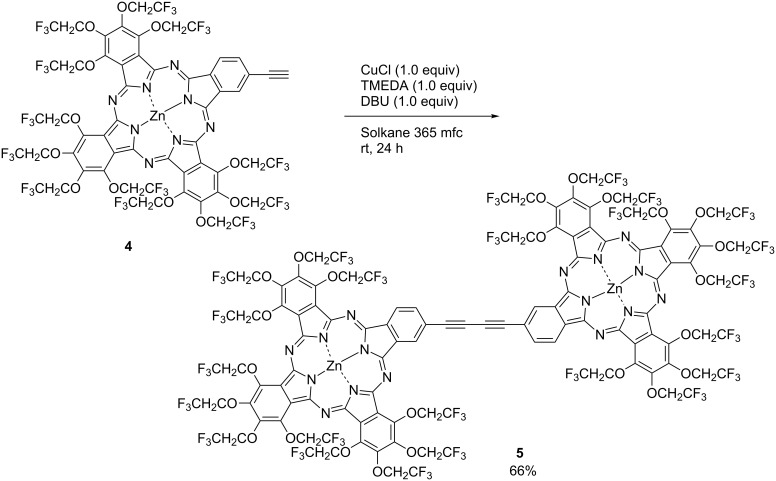
Synthesis of trifluoroethoxy-substituted binuclear phthalocyanine **5** in Solkane^®^ 365 mfc.

TFEO-Pc also shows high solubility in Solkane^®^ 365 mfc, which is a type of fluorine-based solvent. Solkane^®^ 365 mfc is a proven candidate solvent that does not deplete ozone, allowing the use of Solkane^®^ 365 mfc as an alternative to organic solvents to reduce the environmental burden [61–62]. An unsymmetrical type of TFEO-ZnPc with ethynyl group (**4**) shows high solubility in Solkane^®^ 365 mfc while a Glaser-type coupling reaction catalyzed by copper with Solkane^®^ 365 mfc as the medium have been reported (Scheme 2) [63]. In this way, TFEO-Pc can be chemically modified in Solkane^®^ 365 mfc, making it very useful and allowing its expansion to industrial applications.

**Scheme 3:**
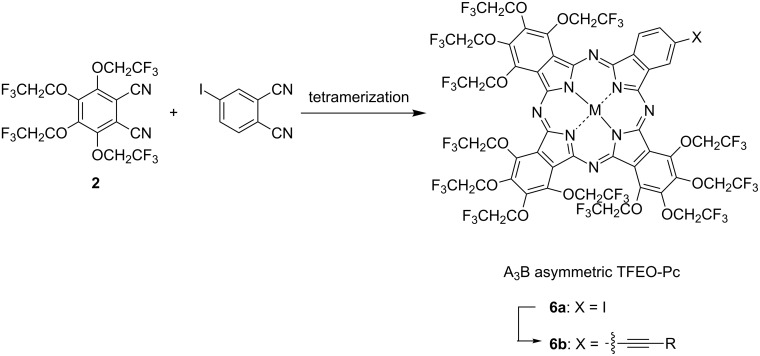
Synthesis of trifluoroethoxy-substituted unsymmetrical phthalocyanines.

Phthalocyanine is usually composed of four isoindoline units. The functionality of phthalocyanine can be enhanced by selectively modifying one of the four units. However, it is difficult to selectively modify the targeted position in the four units using chemical reactions [64–65]. This is because the selective reaction of phthalocyanine is unexplored and removal of byproducts after the reaction is difficult due to its aggregation properties. On the other hand, TFEO-Pcs have high solubility, and chemical modification and purification are easy. For example, the synthesis of unsymmetrical TFEO-Pcs, which are also referred to as A_3_B type phthalocyanines, and their cross-coupling reaction catalyzed by palladium between A_3_B type TFEO-Pcs and acetylenes have been reported (Scheme 3) [66–67]. A_3_B type phthalocyanines are synthesized by mixing two types of phthalonitrile, but symmetric A_4_ and A_2_B_2_ types are simultaneously produced as byproducts. It is usually difficult to remove these symmetrical A_4_ and A_2_B_2_ type Pc isomers from A_3_B type Pc, but TFEO-Pcs can be easily purified by silica gel column chromatography. The extension of π-conjugation by a palladium-catalyzed cross-coupling reaction after a tetramerization reaction proceeds with high yield. Furthermore, since TFEO-Pcs are not only easy to purify but also do not aggregate, their identification by NMR, MS, UV–vis and IR is easy. Consequently, various TFEO-Pcs can be conveniently prepared, setting a path for the development of more functional materials.

### Synthesis of di- and trinuclear trifluoroethoxy-substituted phthalocyanines and investigation of their aggregation properties

**Scheme 4:**
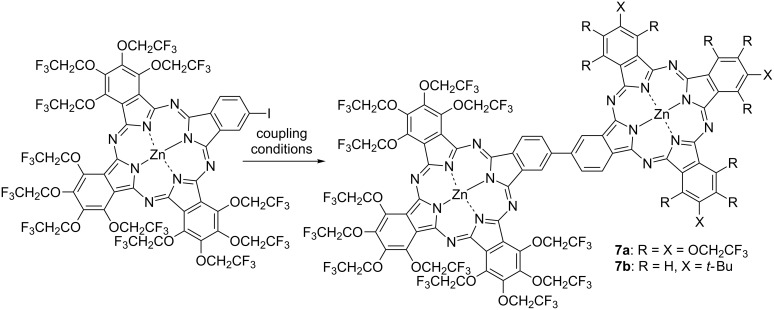
Synthesis of trifluoroethoxy-substituted phthalocyanine dimers linked at the β-position.

**Figure 1:**
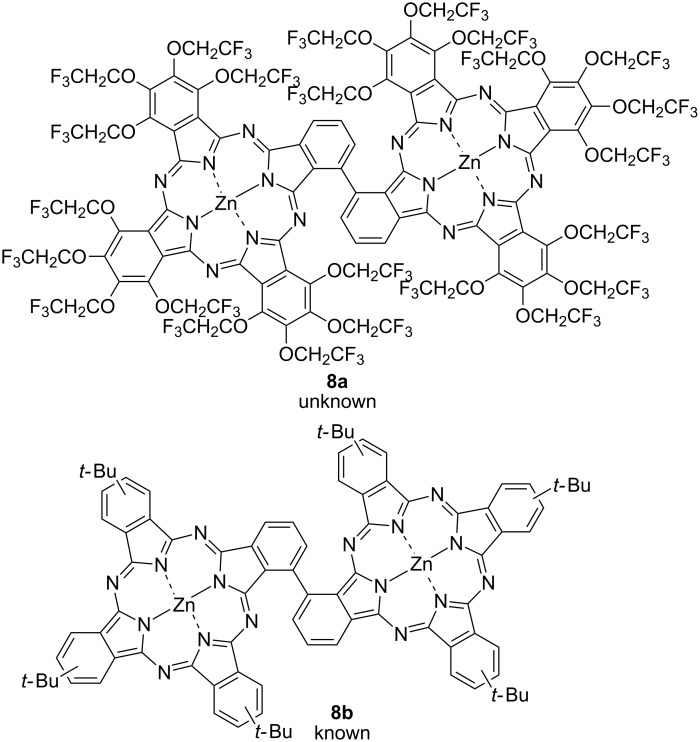
Structure of trifluoroethoxy-substituted phthalocyanine dimers linked at the α-position.

Di- or trinuclear phthalocyanines can be highly functionalized compared with mononuclear phthalocyanines by chemically modifying each unit unsymmetrically caused by an electronic interaction between each unit [68]. In addition, since it is possible to separate charges in dimer-type phthalocyanines, they are expected to be applied to new electron or energy transfer systems [69] such as solar cell materials as well as photocatalysts for organic reactions. A phthalocyanine dimer in which TFEO-Pcs are directly linked by C–C bonds has been reported (Scheme 4) [70–71]. This homodimer was synthesized by a palladium-catalyzed homo-coupling reaction of two A_3_B type monoiodinated TFEO-ZnPcs. There are two positions where the substituent can be introduced into the phthalocyanine, at the α-position near the center of the macrocycle and at the β-position on the outer side of the macrocycle. Even though a TFEO-Pc homodimer connected at the β-position (**7a**) was obtained, the dimer connected at the α-position (**8a**) could not be synthesized due to the repulsion of each unit (Figure 1). Considering that the *tert*-butyl-substituted phthalocyanine dimer connected at the α-position (**8b**) could be synthesized, the repulsive effect of the trifluoroethoxy groups is very strong. Furthermore, the same structural dimer of TFEO-Pc and *tert*-butyl-substituted phthalocyanine **7b** was synthesized by the Suzuki coupling reaction. These dimers show a new absorption peak in the longer wavelength region than the Q band which is not observed in the monomer. This suggests intramolecular electronic coupling between each unit [72]. The authors noted that this intramolecular electronic coupling is likely to be dependent on the overall planarity of the molecules.

**Figure 2:**
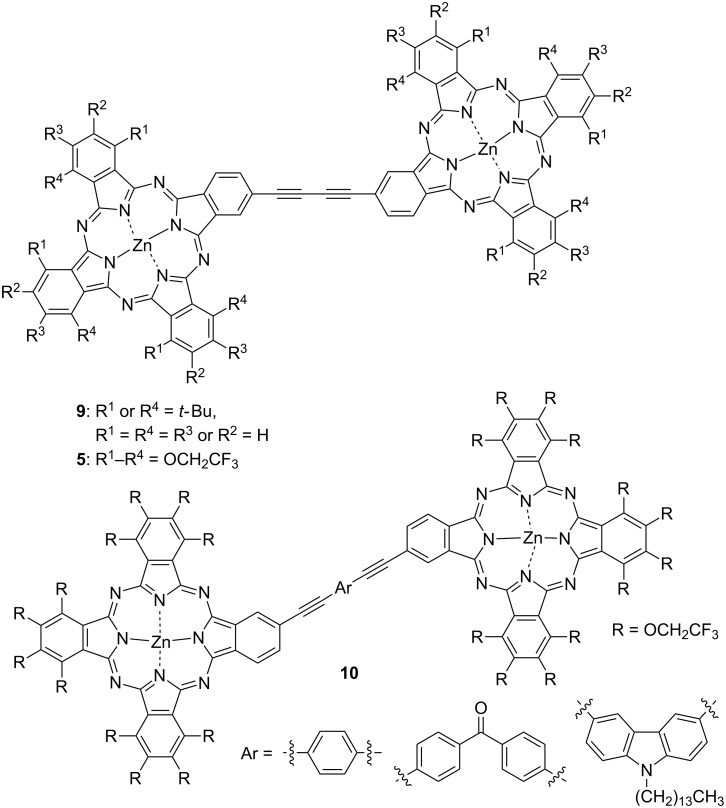
Structure of trifluoroethoxy-substituted dimer via a diacetylene linker.

**Figure 3:**
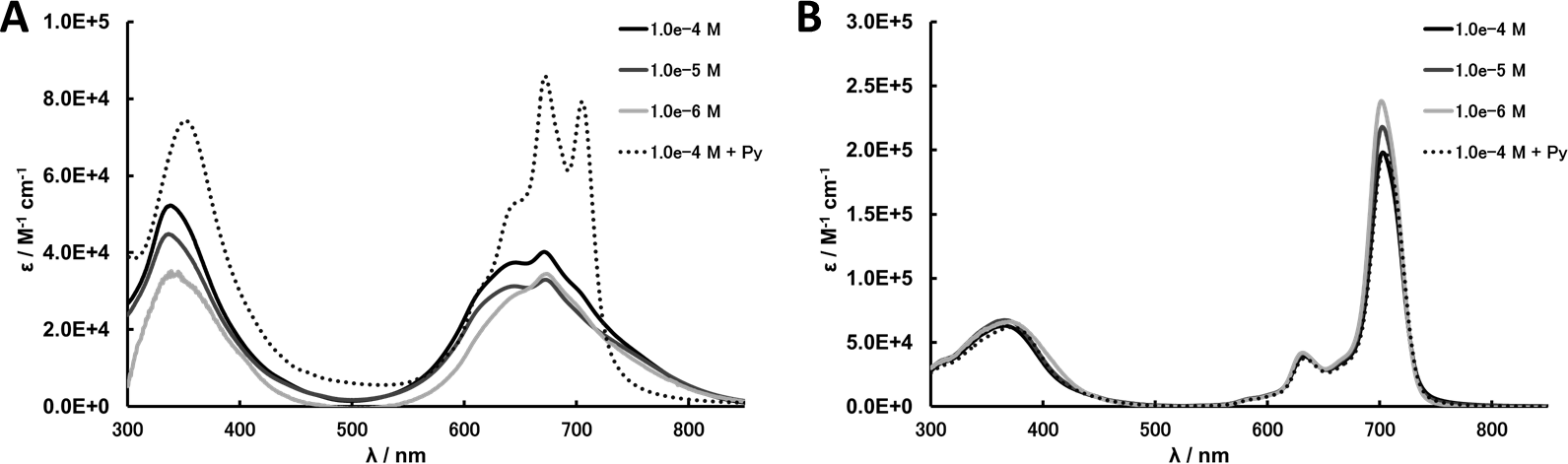
UV–vis spectra of **9** (A) and **5** (B).

Rigid TFEO-Pc dimers with a diacetylene moiety as a linker have also been reported [73–74]. There are reports of dimers via two kinds of diacetylene linkers, via a butadiyne and via an aryldiacetylene moiety (Figure 2). Such binuclear phthalocyanines that are connected via a rigid acetylene linker synthesized by Glaser or Sonogashira reactions have attracted attention due to their interesting effects resulting from further expansion of conjugation. The electronic interaction between the covalently connected chromophore unit leads to important changes in the absorption spectra and so these dimers are expected to serve as attractive building blocks for the construction of multicomponent photoinduced electron transfer supramolecular systems [75–76]. However, phthalocyanine dimers with high rigidity and flatness exhibit a stronger aggregation effect as π-conjugation expands. For example, a *tert*-butyl-substituted phthalocyanine dimer connected via diacetylene linker (**9**) strongly aggregates in solution although *tert*-butyl-substituted mononuclear phthalocyanine does not show any aggregation effect [74]. The aggregation behavior can be estimated from the UV–vis spectra. Phthalocyanine, which is free from aggregation, exhibits a sharp Q band, but the Q band of aggregated phthalocyanine is broadened and shifts to the shorter wavelength region (Figure 3) [77]. The dimer shows a broad Q band in trifluorotoluene which is a typical absorption peak of phthalocyanine aggregates. However, the Q band becomes sharp after the addition of 1% pyridine to the solution [78]. This phenomenon occurs because pyridine coordinates vertically to the central metal of the phthalocyanine and inhibits a stacking interaction between phthalocyanines. Thus, the change in the spectral shape after the addition of pyridine strongly suggests the aggregation behavior of phthalocyanine. On the other hand, binuclear phthalocyanine connected via a diacetylene linker with trifluoroethoxy substituent **5** shows a sharp Q band peak in trifluorotoluene. The absorption spectrum of the dimer does not change even when pyridine is added. This result demonstrates that phthalocyanine does not aggregate in solution.

**Figure 4:**
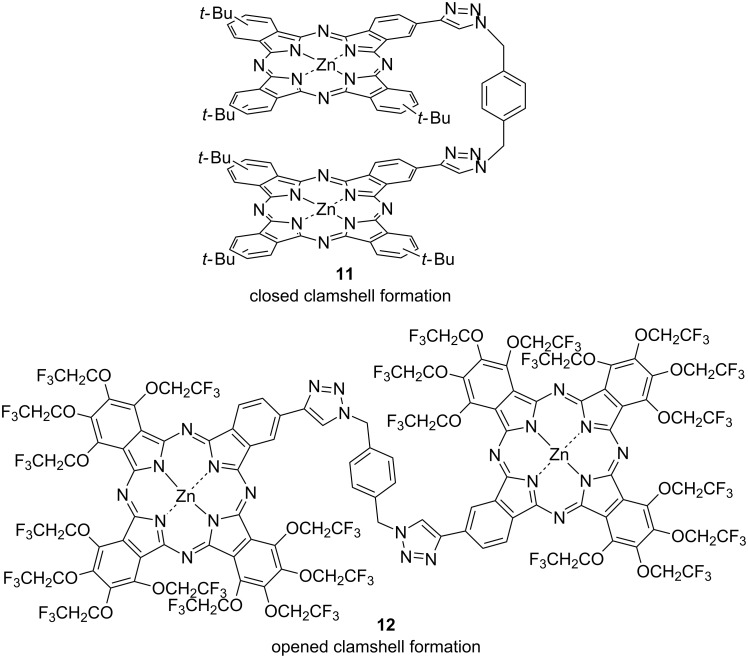
Structure of binuclear phthalocyanines linked by a triazole linker.

**Figure 5:**
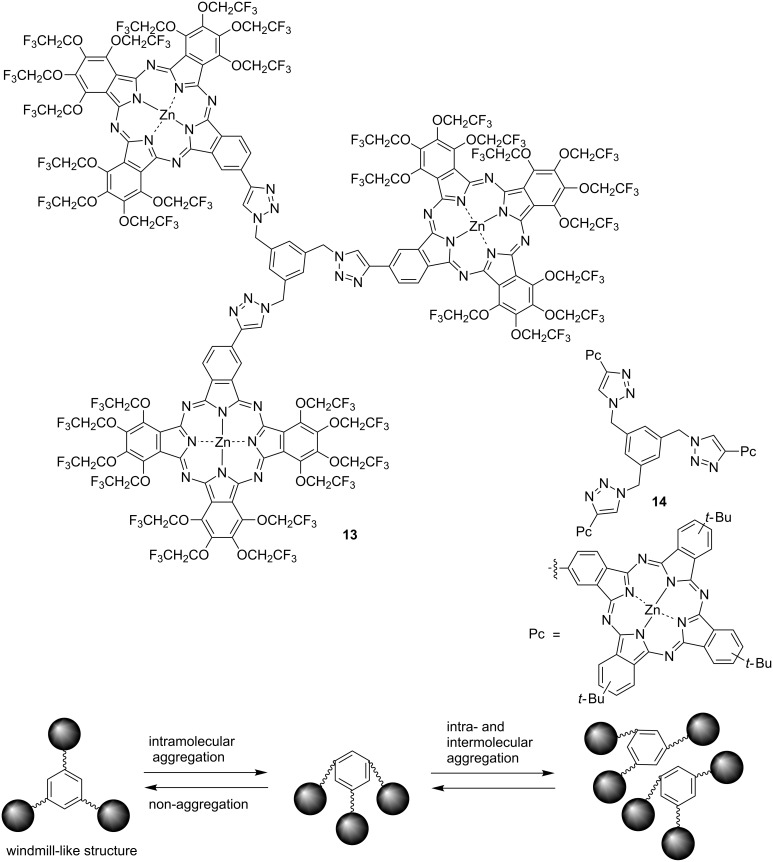
Structure of trinuclear phthalocyanines linked by a triazole linker, and windmill-like molecular structure vs aggregated structure.

It is known that intramolecular aggregation is preferred in binuclear phthalocyanines connected via a flexible linker such as an sp^3^ carbon bond or an ether bond that can rotate freely because of an entropic advantage [79–80]. So these dimers form a folded structure in a so-called closed clamshell conformation. However, not only intermolecular interactions but also intramolecular aggregation can be suppressed due to strong repulsion by the introduction of a trifluoroethoxy group into the flexible binuclear phthalocyanine, which always adopts an open clamshell conformation. Binuclear phthalocyanines connected via a flexible triazole linker that are substituted by a *tert*-butyl (**11**) [81] or trifluoroethoxy (**12**) [82] group have been reported. These dimers were synthesized from A_3_B type phthalocyanines containing an ethynyl group and 1,4-bis(azidomethyl)benzene via a so-called “double-click reaction” [83] catalyzed by CuI. The examination of the spectroscopic properties of these dimers revealed that the *tert*-butyl-substituted phthalocyanine dimer showed a Q band significantly broadened around 700 nm. Furthermore, the shape of the Q band became sharp after the addition of pyridine, as a result of an aggregated dimer caused by an intramolecular stacking interaction and the adoption of a closed clamshell conformation (Figure 4). On the other hand, the trifluoroethoxy-substituted phthalocyanine dimer showed a sharp Q band at around 700 nm. Since there was no change in the spectral structure, even when pyridine was added, this dimer always assumed an opened clamshell conformation caused by the repulsive effect of the trifluoroethoxy group. More interestingly, similar aggregation behaviors were also suggested for trinuclear phthalocyanines that can aggregate more easily [84]. These trinuclear phthalocyanines were synthesized by a triple click reaction. The *tert*-butyl-substituted trimer **14** exhibited aggregation behavior by an intramolecular stacking interaction between the units (Figure 5). On the other hand, the trifluoroethoxy-substituted trimer **13** did not exhibit such aggregation behavior and adopted a “windmill-like molecular structure” by the repulsive action of the trifluoroethoxy group. Thus, it was found that not only the binuclear phthalocyanine connected via a flexible linker but also the trinuclear phthalocyanine can completely suppress the intermolecular stacking interaction by introduction of a trifluoroethoxy group into the periphery of phthalocyanine.

### Application of trifluoroethoxy-substituted phthalocyanines to biology

**Scheme 5:**
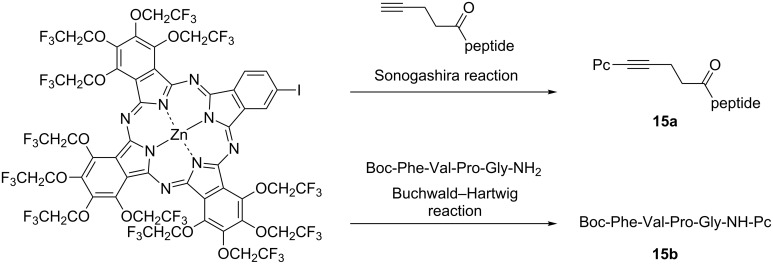
Synthesis of trifluoroethoxy-substituted phthalocyanines conjugated with peptides.

Phthalocyanines are expected to be applied to medical fields [85–86] such as biological image probes and as agents for photodynamic therapy because of their excellent spectroscopic properties, allowing phthalocyanine to be a useful medical dye because it absorbs light of long wavelengths with high tissue transparency. However, ordinary phthalocyanines cause the deterioration of spectroscopic properties and solubility by forming aggregates, so it is necessary to suppress this aggregation [87]. As described above, the ability of the trifluoroethoxy group in suppressing the aggregation of phthalocyanine is extremely remarkable. Therefore, it is expected that an effective medical dye can be developed by using this excellent aggregation suppressing effect. On the other hand, improving the biocompatibility of phthalocyanine is as important as suppressing its aggregation property. It is also necessary to improve the water solubility of the lipophilic phthalocyanine in order to increase its biocompatibility [88–89]. One frequent method to increase the water solubility of phthalocyanine involves the combination of a biomolecule such as a sugar [90–91] or a peptide [92]. For example, TFEO-Pcs conjugated with peptides (**15**) have been reported [93]. A_3_B type TFEO-Pc was successfully condensed with peptides by palladium-catalyzed cross-coupling reactions in good yield (Scheme 5). These TFEO-Pc-peptide conjugates, which show a sharp Q band in the UV–vis spectrum, do not aggregate in solution. These dyes are expected to be developed into medical imaging probes.

**Scheme 6:**
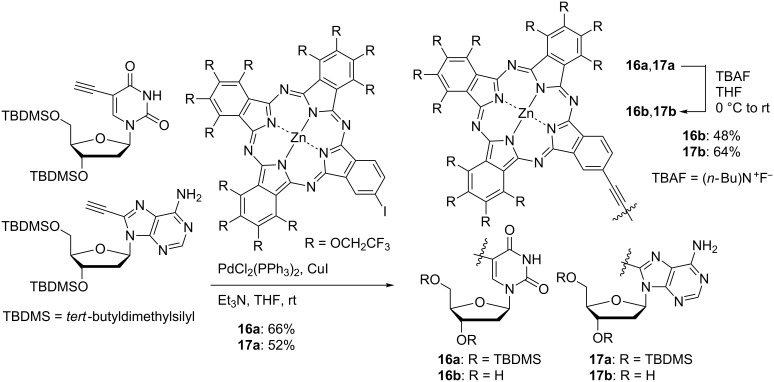
Synthesis of trifluoroethoxy-substituted phthalocyanines conjugated with deoxyribonucleosides.

Phthalocyanine is expected to be used not only as a biological imaging agent but also in cancer treatment. Photodynamic therapy (PDT) is a laser cancer therapy that introduces organic dyes into cancer cells and kills them by reactive oxygen species with cytotoxicity generated by light irradiation [94–95]. In addition to reducing the psychological burden of the patients because this process does not require surgical operation, damage to normal cells can be minimized by irradiating the laser only to the target tumor cells. The features of ideal PDT agents include, among others: i) strong absorption in the red wavelength region; ii) non-aggregation property; iii) high biocompatibility; iv) good selectivity to cancer cells; v) high quantum yield of singlet oxygen [96]. Since the PDT activity of phthalocyanine is remarkably decreased by aggregation, TFEO-Pc, which has a non-aggregation property, is expected to be a highly active PDT drug. Incidentally, improving drug selectivity for cancer cells is a problem in developing cancer therapeutic drugs. Phthalocyanines have the capacity to accumulate in cancer cells due to the EPR (enhanced permeation and retention) effect [97–98]. This is a phenomenon in which new blood vessels around cancer cells allow macromolecules to pass easily through cells, causing the accumulation of molecules around cancer cells as a result. This effect is more likely to occur with a long residence time of the compound in blood. The residence time in blood is prolonged by increasing the water solubility of the drug. An ideal PDT drug would improve the water solubility of TFEO-Pc. In 2006, TFEO-ZnPcs were conjugated with deoxyribonucleosides **16** and **17** for possible use in PDT [99]. The deoxyribonucleoside unit is expected to enhance the water solubility of the phthalocyanine, facilitating its incorporation into cancer cells which undergo active cell division. These TFEO-ZnPc/deoxyribonucleoside hybrids were synthesized from unsymmetrical A_3_B-type TFEO-ZnPc under Sonogashira cross-coupling conditions (Scheme 6). UV–vis absorption measurements suggested that the deoxyribonucleoside-linked TFEO-Pcs had a non-aggregation property, as expected. Unfortunately, it was also found that these conjugates gradually decomposed under light irradiation, so their application as an anticancer agent was discontinued.

**Scheme 7:**
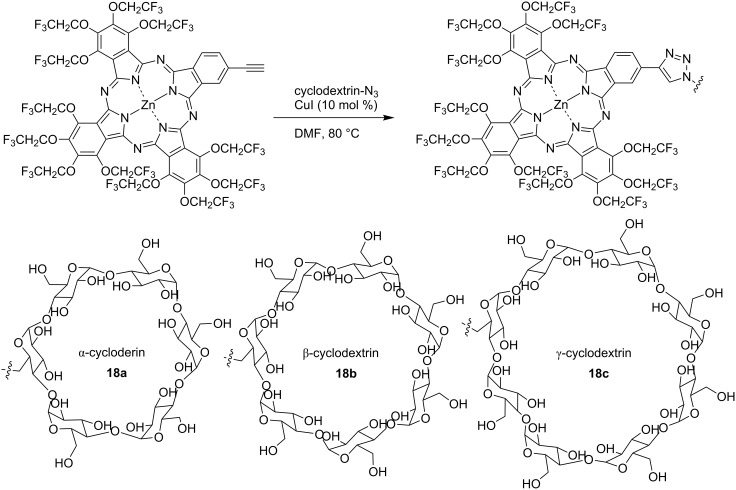
Synthesis of trifluoroethoxy-substituted phthalocyanines conjugated with cyclodextrin.

After that TFEO-ZnPc/cyclodextrin conjugates **18** were reported under similar conditions (Scheme 7) [100]. That study revealed that cyclodextrin-linked TFEO-Pcs have a non-aggregation property and sufficient water solubility, similar to the deoxyribonucleoside conjugates. An in vitro investigation showed that cyclodextrin-linked TFEO-Pcs were ideal PDT drugs that exhibits high cytotoxicity under light irradiation while displaying little cytotoxicity in the dark (Table 2). On the other hand, interestingly, the fluorine-free *tert*-butylated derivative showed cytotoxicity even in the dark. This result suggests that the trifluoroethoxy group reduces the cytotoxicity of the phthalocyanine. Subsequently, an in vivo investigation was also conducted. Chicken embryos transplanted with cancer cells were treated with PDT by a cyclodextrin conjugate and proved 17 days later. The cancer cells of embryos treated with PDT had shrunk to about half the size of untreated embryos. On the other hand, when cancer cells were not irradiated with light after administration of the cyclodextrin conjugate, these cells were almost of the same size as at unprocessed embryos. These results suggest that the cyclodextrin conjugate does not display cytotoxicity when not irradiated with light. Therefore, the cyclodextrin conjugate has the potential to be effective and very safe.

**Table 2:** Comparison of IC_50_ values with laser PDT among phthalocyanine conjugated with cyclodextrin against B16-F10.

	IC_50_ (μM)		
	
compound	drug only	PDT^a^	effective ratio^b^

**19**: β-CD-*t*-Bu-Pc	16.7	1.10	15.2
**18a**: α-CD-TFEO-Pc	≥50	1.29	≥38.8
**18b**: β-CD-TFEO-Pc	≥50	1.12	≥44.6
**18c**: γ-CD-TFEO-Pc	≥50	1.25	≥40.0

^a^PDT comprised laser irradiation. ^b^Effective ratio shows ratio vs drug only.

### Application of trifluoroethoxy-substituted phthalocyanines to functional materials

#### Development for organic thin-film solar cells

**Figure 6:**
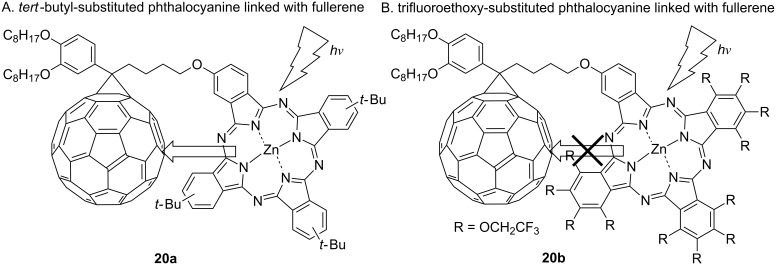
Direction of energy transfer of phthalocyanine–fullerene conjugates.

The introduction of trifluoroethoxy groups imparts not only a non-aggregation property and high solubility but also an electron-deficient π space and thermal or chemical stability to the phthalocyanine. For these reasons, it is expected that TFEO-Pcs will be developed for the application in functional dyes with higher performance and in new industrial fields. An interesting example involves investigations on energy transfer between the phthalocyanine and the fullerene [101]. Normally, phthalocyanine plays the role as a donor type molecule in the field of organic semiconductors [102]. For example, a hybrid compound consisting of phthalocyanine and fullerene **20a** causes the transfer of energy from the phthalocyanine unit to the fullerene unit when irradiated by light (Figure 6) [103–104]. However, such energy transfer does not occur in the hybrid of TFEO-ZnPc and fullerene **20b** because the strong electron-withdrawing property of fluorine reverses the relationship between the electronic states of both units [105]. It was suggested that TFEO-Pc has an orbital energy closer to that of [6,6]-phenyl-C_61_-butyric acid methyl ester (PCBM) [106–107], which is a known acceptor molecule, than to poly(3-hexylthiophene-2,5-diyl) (P3HT) [107], which is a known donor molecule. It can thus be concluded that TFEO-Pc can play a role as an acceptor-type semiconductor. In general, organic semiconductors have a greater abundance of donor-type molecules than acceptor-type molecules. Therefore, the structural development of acceptor molecules is restricted. TFEO-Pcs could be developed as a new acceptor-type molecule.

In fact, solar cells that utilize the ability of TFEO-Pc to accept energy have been developed. A planar heterojunction organic thin-film solar cell was prepared by using P3HT as the donor and TFEO-ZnPc as the acceptor [108–109]. Photoelectric conversion was observed in a wide absorption region, suggesting that TFEO-Pcs might be used as a substitute for fullerene in solar cells. In addition, the influence of the number of introduced trifluoroethoxy groups on the performance of solar cells has been investigated. The open circuit voltage (*V_OC_*), short circuit current density (*J_SC_*) and fill factor (FF) of heterojunction solar cells using various TFEO-Pcs were estimated and their conversion efficiency (*E_ff_*) was compared (Table 3). The solar cell that used phthalocyanine with a single trifluoroethoxy group introduced at the β-position, rather than trifluoroethoxy full-coated phthalocyanine, showed the best performance. In the development of organic thin-film solar cells, dye material needs a moderate aggregation property for effective energy transfer [110]. Therefore, it is considered that the performance of solar cells that use per(trifluoroethoxy)phthalocyanine, which exhibits a strong repulsion effect, will be low. It is expected that phthalocyanine will be put to practical use within solar cells by improving the orientation control in thin films.

**Table 3:** Characteristics of phthalocyanine/P3HT heterojunction organic thin-film solar cells.

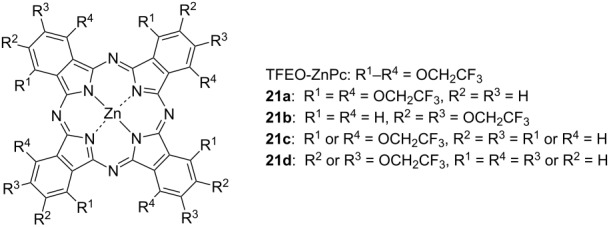				

	*E*_ff_ (%)	FF	V_oc_ (V)	*J*_sc_ (mA/cm^2^)

P3HT only	2.4 × 10^−3^	0.41	0.22	2.7 × 10^−2^
TFEO-ZnPc	3.3 × 10^−2^	0.29	0.24	0.48
**21a**	6.8 × 10^−3^	0.26	0.21	0.13
**21b**	8.1 × 10^−5^	0.15	0.53	1.1 × 10^−3^
**21c**	1.2 × 10^−2^	0.32	0.35	0.10
**21d**	7.1 × 10^−2^	0.38	0.42	0.45

#### Development for fluorinated polymer dyes

**Scheme 8:**
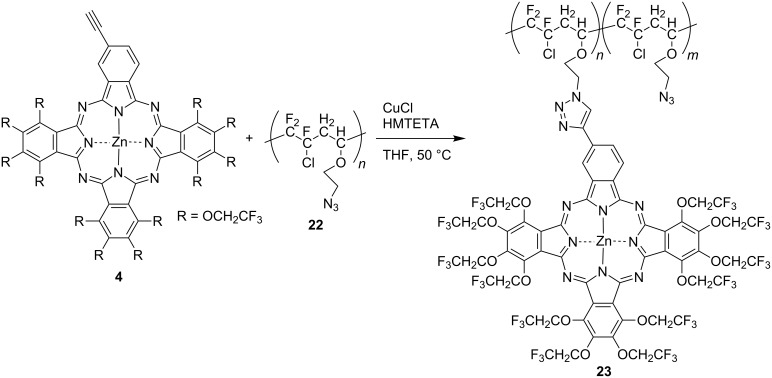
Synthesis of fluoropolymer-bearing phthalocyanine side groups.

Fluoropolymers are widely used from household products to aerospace materials because they show excellent stability, as well as non-adhesive, electrical and optical properties due to the specific properties of fluorine [24,111]. Material development studies of compounds that combine the properties of fluoropolymers and the spectroscopic properties of phthalocyanines have been conducted [112–113]. TFEO-Pc-supported fluoropolymers **23** that combine the properties of a fluorinated polymer and the spectroscopic properties of a phthalocyanine have been developed [114]. A_3_B TFEO-ZnPc **4** and fluorinated polymer **22** were condensed by a click reaction between azide and alkyne groups (Scheme 8). The graft ratio of the TFEO-ZnPc-supported fluoropolymer was calculated from the area ratio of ^19^F NMR, and the resulting fluorinated copolymers showed different grafting ratios (from 10 to 72%) depending on the reaction conditions. A thermogravimetric analysis showed a weight loss of more than 90% at 210 °C for the precursor before condensation of TFEO-ZnPc, whereas the TFEO-ZnPc-supported fluoropolymer showed only 5% weight loss at 300 °C. It was suggested that introduction of TFEO-ZnPc into a fluoropolymer markedly improves its thermal stability. In addition, the TFEO-ZnPc-supported fluoropolymer showed significantly lower fluorescence than monomeric TFEO-ZnPc. This suggests a strong electronic interaction among TFEO-Pc units, allowing for its application in photonic devices.

### Synthesis of double-decker phthalocyanines and investigation of the repulsive effect of trifluoroethoxy substituents

**Scheme 9:**
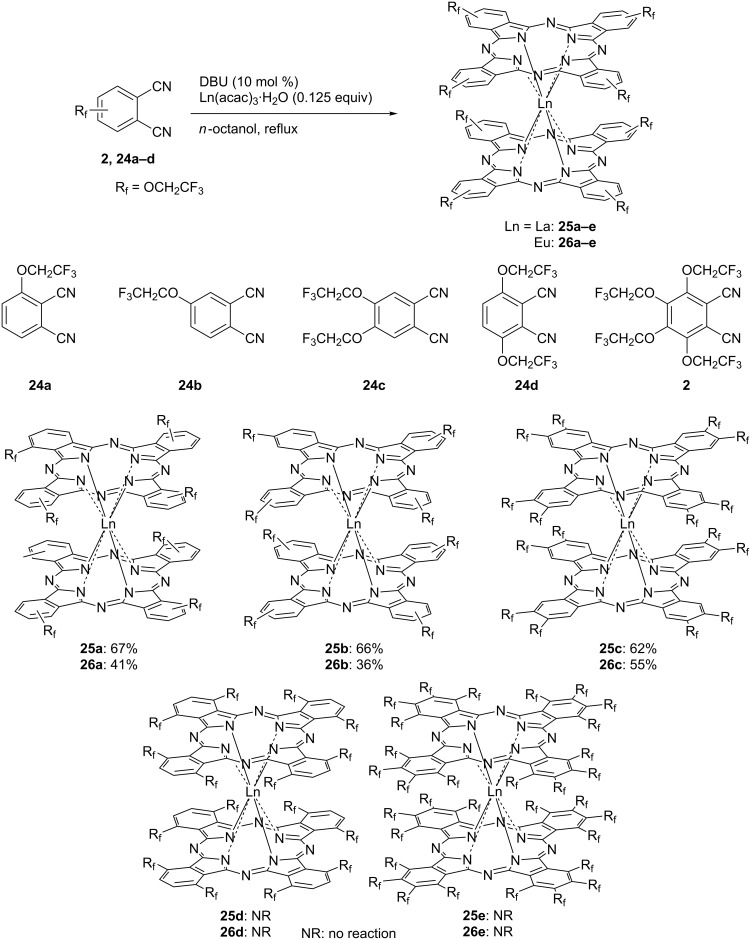
Synthesis of trifluoroethoxy-substituted double-decker type phthalocyanines.

The properties of trifluoroethoxylated phthalocyanine depend on the number of substituted trifluoroethoxy groups and their substitution positions. Various trifluoroethoxylated phthalocyanines having a different number of substituents and substituted positions have been reported [115–118]. The effect of the substitution of the trifluoroethoxy groups on the aggregation properties has been investigated by a synthetic study of trifluoroethoxy-substituted double-decker type phthalocyanines **25** and **26** [119]. When a rare earth element is used as the central metal of the phthalocyanine, it is known that phthalocyanine macrocycles form a double-decker phthalocyanine (DDPc) in which two macrocycles are coordinated above and below the metal [120–121]. The double-decker structure can be regarded as a phthalocyanine which forcibly formed an aggregate by a rare earth element. Therefore, when TFEO-Pc showing a non-aggregation property by a strong repulsion effect is used, it is interesting to know whether the formation of DDPc is possible or not. First, it was investigated whether the phthalocyanine can be synthesized by using phthalonitrile in which one trifluoroethoxy group was introduced at the α- or β-position (Scheme 9). The target DDPc was successfully obtained in moderate yield by heating trifluoroethoxy-substituted phthalonitrile in the presence of DBU and a lanthanoid acetylacetone complex in *n*-octanol. Next, the synthesis of DDPc using a phthalonitrile having two trifluoroethoxy groups at the α- or β-position was investigated. Even though, β-disubstituted trifluoroethoxylated DDPcs were obtained with the same yield as the mono-substituted product. The reaction did not proceed in the case of the α-disubstituted product. Furthermore, DDPc could also not be synthesized using phthalonitrile in which all four positions were trifluoroethoxylated. These results suggest that a trifluoroethoxy group substituted at the α-position has a greater repulsion effect than substituted at the β-position of the phthalocyanine. In the case of α-disubstituted phthalocyanine, it is believed that the repulsion effect is too high to obtain a double-decker type structure. Among the DDPcs obtained, β-trifluoroethoxy-substituted DDPcs showed a strong aggregation behavior despite the presence of pertrifluoroethoxy substitutions.

### Synthesis and optical properties of trifluoroethoxy-substituted subphthalocyanines

**Scheme 10:**
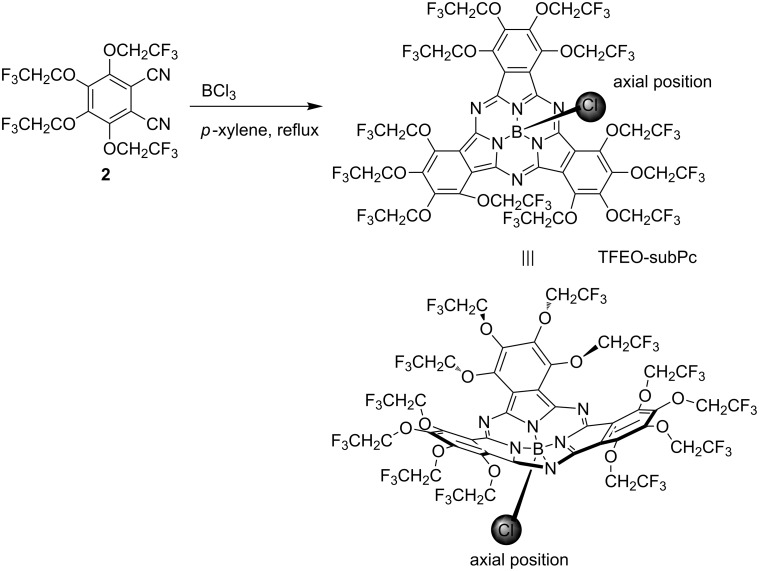
Synthesis of trifluoroethoxy-substituted subphthalocyanine.

Subphthalocyanines [122–125] are phthalocyanine analogues unlike phthalocyanines which are planar compounds composed of four isoindoline units, and are cone-shaped distorted molecules composed of three isoindoline units. The only central element of subphthalocyanine is boron, and it has a ligand in the axial direction on the boron. Since subphthalocyanines have excellent spectroscopic properties and a unique three-dimensional structure, it is expected that they will be applied to various fields [126–128], alongside phthalocyanines. However, the solubility of subphthalocyanines is not very high despite of the non-aggregation phenomenon due to their three-dimensional structure. On the other hand, since trifluoroethoxy-substituted subphthalocyanines (TFEO-subPcs) have high solubility due to the influence of fluorine and are easy to handle, it is expected that various derivatives as well as TFEO-Pcs will be developed. TFEO-subPc is synthesized by cyclotrimerisation of tetrakis(trifluoroethoxy)phthalonitrile (**2**) in the presence of boron trichloride in *p*-xylene under reflux conditions (Scheme 10) [129]. There are two methods to synthesize subphthalocyanine derivatives, mainly peripheral functionalization and substitution of axial positions. Among them, substitution reactions at the axial position can easily deliver many subphthalocyanine derivatives by coordinating various chemical species with one subphthalocyanine as a foothold. However, it is known that axial substitution reactions proceed with low yield with ordinary subphthalocyanine [130]. On the other hand, TFEO-subPc remarkably improves the substitution activity at its axial position, and the axial substitution reaction of alcohols proceeds with high yield under the classical reaction conditions of heating in the presence of triethylamine in toluene. Unsubstituted subphthalocyanine (H-subPc) and perfluorinated subphthalocyanine (F-subPc) were also synthesized, and the efficiency of the axial substitution reaction with triethylene glycol was compared (Table 4). The yield of the substitution reaction of H-subPc was only 5%, and the reaction did not proceed in the case of F-subPc. On the other hand, in TFEO-subPc, the substitution reaction at the axial position proceeded successfully, and the target substitution was obtained at a rate of 68%. This is because the stability of subphthalocyanine and the reactivity of the substitution reaction at the axial position are improved by the introduction of a trifluoroethoxy group.

**Table 4:** Comparison of axial ligand substitution reactivity of subphthalocyanines.

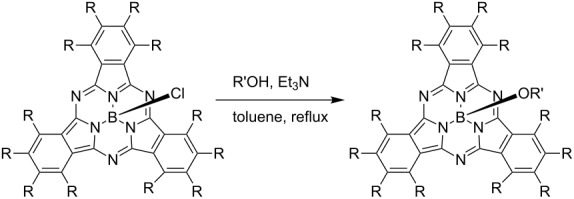				

entry	subPc	R	R’OH	yield (%)

1	H-subPc	H	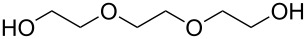	5
2	F-subPc	F		NR
3	TFEO-subPc	OCH_2_CF_3_		68

**Figure 7:**
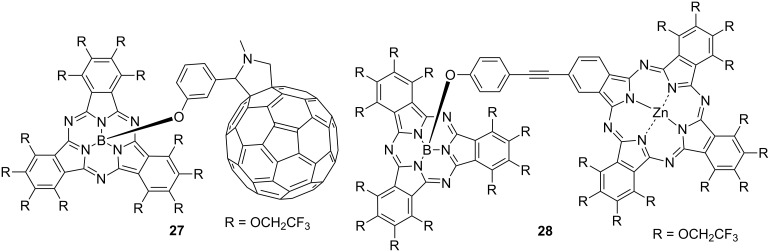
Structure of axial ligand substituted subphthalocyanine hybrid dyes.

Using this high reactivity of axial substitution, hybrid dyes in which the axial position of TFEO-subPc is substituted with fullerene **27** or phthalocyanine **28** intended to construct energy transfer systems have been synthesized (Figure 7). In the previous section, it was explained that the hybrid dye of TFEO-ZnPc and fullerene does not cause energy transfer between units, but in this case, the TFEO-subPc unit acts as a donor.

**Scheme 11:**
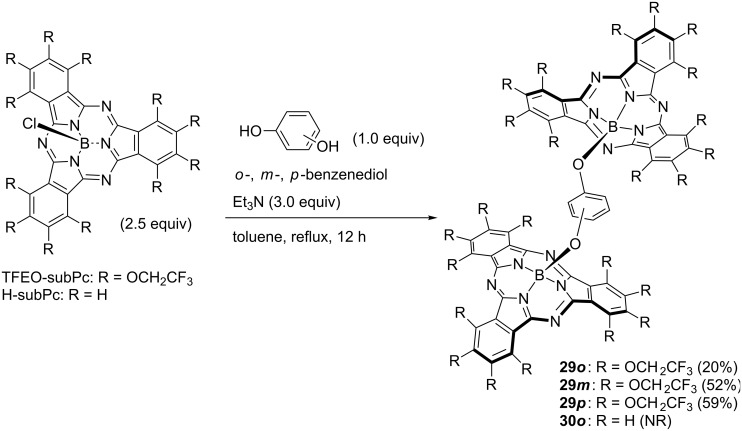
Synthesis of subphthalocyanine homodimers.

**Scheme 12:**
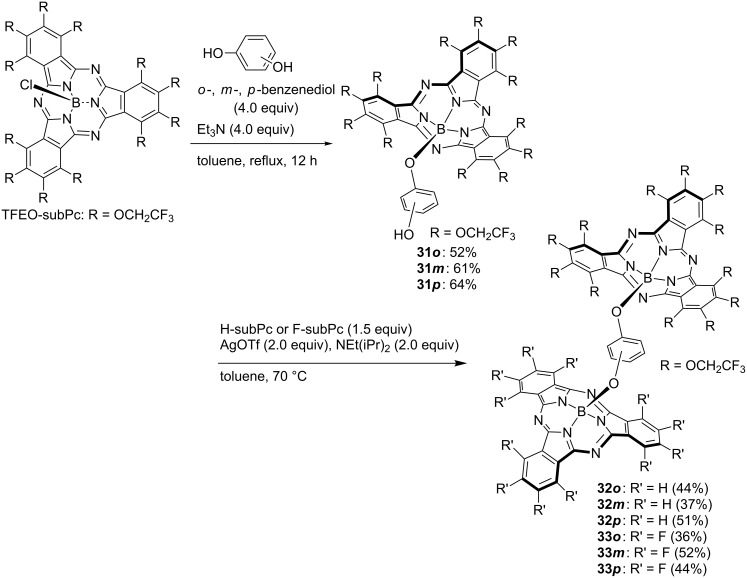
Synthesis of subphthalocyanine heterodimers.

**Figure 8:**
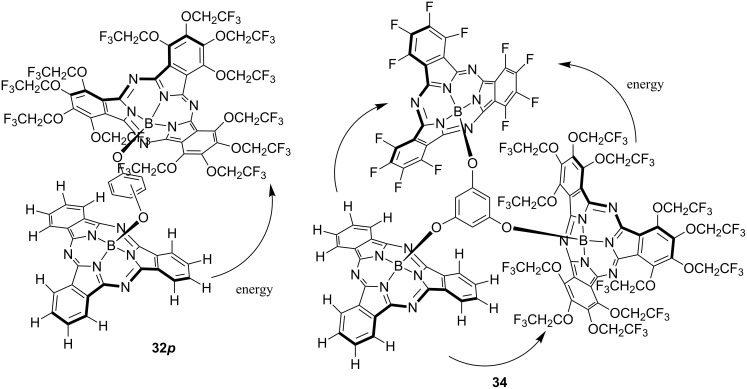
Energy transfer between subphthalocyanine units.

A composite of subphthalocyanine was synthesized by using the high axial position substitution activity of TFEO-subPc. TFEO-subPc homodimers **29** substituted at the axial position in the *ortho*-, *meta*-, or *para*-position were synthesized by reacting TFEO-subPc with catechol, resorcinol or hydroquinone in toluene in the presence of triethylamine (Scheme 11) [131]. The synthesis of H-subPc homodimer **30** was attempted using the same method, but the target substance was obtained. This is a good example showing the high activity of the axial substitution reaction of TFEO-subPc. The synthesis of the heterodimer of TFEO-subPc with H-subPc or F-subPc was also attempted. However, since the substitution activity on the axial position of H-subPc and F-subPc was unsatisfactory, the reaction did not progress as expected. Therefore, the activation method for the axial position using trifluoromethanesulfonate reported by Torres et al. was investigated [132]. The reaction proceeded successfully and the desired heterodimers **32** and **33** were obtained (Scheme 12). The investigation of these spectroscopic properties revealed that energy transfer occurs between units in the TFEO-subPc and H-subPc heterodimer. Since the UV–vis spectra of a heterodimer are the sum of the absorption spectra of the respective units, it is suggested that each unit has an independent π-conjugate system. On the other hand, the fluorescence quantum yield decreased as a whole and the fluorescence derived from H-subPc was remarkably quenched. These results suggest energy transfer from the H-subPc unit to the TFEO-subPc unit (Figure 8) [133]. Subsequently, an unsymmetrical subphthalocyanine trimer composed of TFEO-subPc, F-subPc and H-subPc with phloroglucinol as a linker by the axial substitution reaction, has also been reported [134]. The synthesis of this trimer was carried out by the following sequential method. First, the axial position of TFEO-subPc-Cl was substituted with phloroglucinol under reflux conditions in toluene in the presence of triethylamine. Next, the connection of F-SubPc under reflux conditions was attempted, but the reaction did not proceed because of the poor axial reactivity. Therefore, the axis substitution reaction for F-SubPc was carried out after activating its axial position by silver triflate to obtain an intermediate dimer. Finally, H-SubPc was condensed by the axial substitution reaction using the activation method by silver triflate to induce the desired trimer **34**. Even in the trimer, an investigation of the spectroscopic properties suggested the transfer of energy between units. Each unit had an independent absorption property, but the fluorescence peak corresponding to H-subPc was quenched. This result suggests energy transfer from the H-subPc unit to fluorine-containing units. The relationship of HOMO and LUMO energy levels of SubPcs estimated from electrochemical measurements and DFT calculations is as follows: H-SubPc > TFEO-SubPc > F-SubPc. From these results, energy transfer between units is assumed as three patterns: from H-subPc to the TFEO-subPc or F-subPc unit, and from TFEO-subPc to the F-subPc unit.

### Synthesis and optical investigation of trifluoroethoxy-substituted benzene-fused phthalocyanines

**Figure 9:**
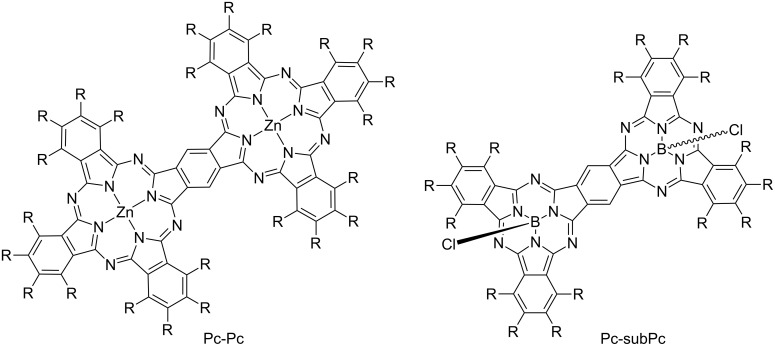
Structure of phthalocyanine and subphthalocyanine benzene-fused homodimers.

**Scheme 13:**
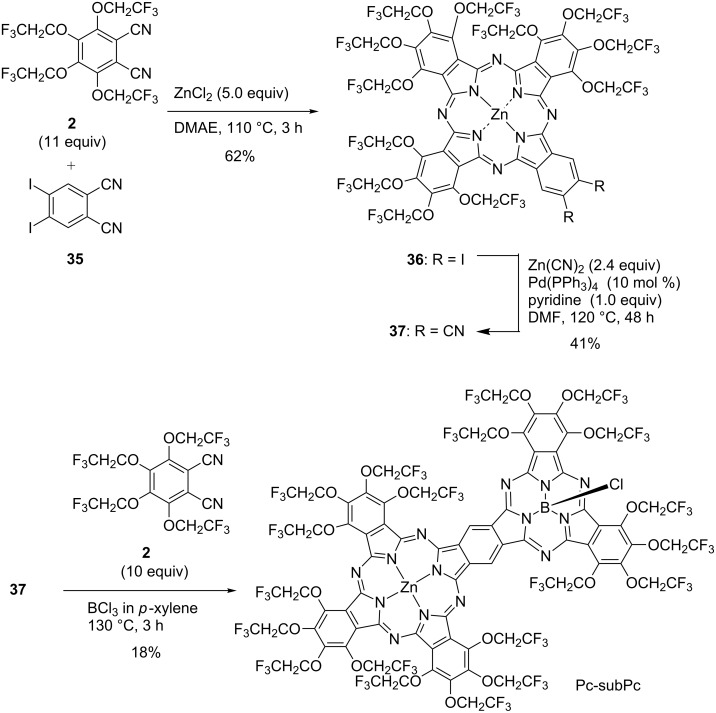
Synthesis of a phthalocyanine and subphthalocyanine benzene-fused heterodimer.

**Figure 10:**
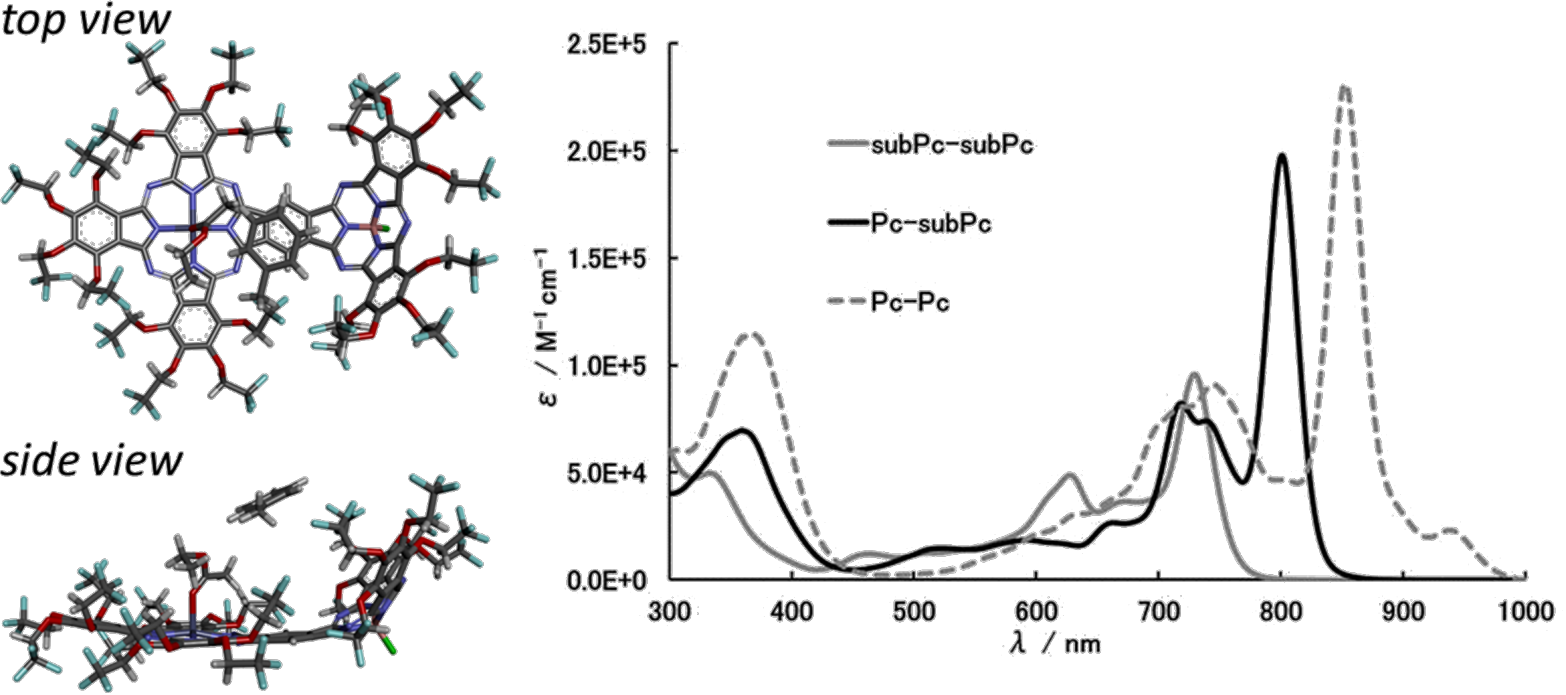
X-ray crystallography of Pc-subPc (left) and UV–vis spectra of benzene-fused dimers.

One of the binuclear phthalocyanines that have attracted attention in recent years is the benzene-fused phthalocyanine dimer in which two phthalocyanines are fused together, having a common benzene ring. The benzene-fused dimer shows strong absorption in the near infrared region due to its large conjugated planar π-systems, and exhibits interesting features such as a strong electronic interaction between each unit through a common benzene ring. The benzene-fused phthalocyanine dimer was first reported in 1987, and various benzene-fused dimer derivatives have been reported ever since [135–136]. Although various benzene-fused phthalocyanine dimers (Pc-Pcs) and benzene-fused subphthalocyanine dimers (subPc-subPcs) have been reported (Figure 9) [137–140], there is only one report of a benzene-fused phthalocyanine and a subphthalocyanine heterodimer (Pc-subPc). In 2014, a trifluoroethoxy-substituted Pc-subPc was reported and its properties were compared with homodimers [141]. This heterodimer was synthesized by a stepwise synthesis method in which a phthalocyanine unit was first synthesized and then a subphthalocyanine unit was formed (Scheme 13). The synthesis of Pc-subPc was carried out as follows. First, A_3_B type diiodophthalocyanine **36** was synthesized by an unsymmetrical synthesis using diiodophthalonitrile **35** and trifluoroethoxyphthalonitrile **2**, and then iodides were converted to cyano groups by a coupling reaction. Finally, dicyanophthalocyanine **37** was reacted with **2** in the presence of boron trichloride to obtain trifluoroethoxy-substituted Pc-subPc. Although Pc-subPc was synthesized via a two-step statistical unsymmetric synthesis, it can be easily purified by silica gel column chromatography due to the aggregation inhibitory effect caused by the trifluoroethoxy group. The structure of Pc-subPc was confirmed by X-ray crystal structural analysis, having both a planar structure derived from phthalocyanine and a cone-shaped structure derived from subphthalocyanine (left of Figure 10). In the UV–vis absorption spectrum, Pc-subPc shows an absorption band around 800 nm which is located between the absorption bands of Pc-Pc and subPc-subPc (right of Figure 10). This result is consistent with a decrease in the HOMO–LUMO gap due to the conjugate expansion of each dimer, and it can be said that Pc-subPc is a dye showing intermediate spectroscopic properties between Pc-Pc and subPc-subPc.

## Conclusion

This review is a summary of research on trifluoroethoxy-substituted phthalocyanines and subphthalocyanines. Due to the specific properties of fluorine, the aggregation behavior of phthalocyanine is alleviated, and various beneficial properties such as solubility and spectroscopic properties are enhanced. Since trifluoroethoxy-substituted phthalocyanines and subphthalocyanines are easy to handle, it is possible to obtain diverse derivatives and to fully demonstrate their functions. They are also expected to be developed into new material fields due to their high stability, high solubility and high lipophilicity. By combining the characteristics of fluorine and the functionalities of phthalocyanine, it is expected that new applications of phthalocyanines can be explored in furture.
